# Ligand‐Directed Template Assembly for the Construction of Gigantic Molybdenum Blue Wheels†

**DOI:** 10.1002/anie.201901818

**Published:** 2019-06-28

**Authors:** Weimin Xuan, Robert Pow, Qi Zheng, Nancy Watfa, De‐Liang Long, Leroy Cronin

**Affiliations:** ^1^ School of Chemistry The University of Glasgow Glasgow G12 8QQ UK

**Keywords:** anion templation, gigantic cluster, host–guest chemistry, molybdenum blue, polyoxometalates

## Abstract

Template‐mediated synthesis is a powerful approach to build a variety of functional materials and complex supramolecular systems. However, the systematic study of how templates structurally evolve from basic building blocks, and then affect the templated self‐assembly, is critical to understanding and utilizing the underlying mechanism, to work towards designed assembly. Here we describe the templated self‐assembly of a series of gigantic Mo Blue (MB) clusters **1**–**4** using l‐ornithine as a structure‐directing ligand. We show that by using l‐ornithine as a structure director, we can form new template⊂host assemblies. Based on the structural relationship between encapsulated templates of {Mo_8_} (**1**), {Mo_17_} (**2**) and {Mo_36_} (**4**), a pathway of the structural evolution of templates is proposed. This provides insight into how gigantic Mo Blue cluster rings form and could lead to full control over the designed assembly of gigantic Mo‐blue rings.

Template‐mediated synthesis is a versatile approach to fabricate nanostructured materials and supramolecular architectures.^1^ Anion templates are widely investigated in biological systems,^2^ but they are much less explored in supramolecular inorganic chemistry, in comparison to cationic and neutral templates.^3^ In the field of polyoxometalates (POMs)^4^—a unique class of anionic discrete metal–oxo clusters with a wide variety of structures and properties—anion templates are essential for their construction.^5^, ^6^, ^7^ Not only do these anions direct the self‐assembly of POMs via a templating effect but also affect the resultant structures and their properties.^8^ More recently, nanosized POM clusters have been found as anion templates to direct the self‐assembly of larger POMs.^9^ One example is the formation of {Mo_36_} upon acidification of molybdate, which allows for the self‐assembly of the {Mo_154_} giant Mo Blue wheel after addition of the reducing agent, revealing the role of the {Mo_36_} anion template.9a In general, three types of interactions can be established between the templates and POMs: hydrogen bonding,9c electrostatic interactions9a and coordination,9f see Scheme 1.9d To exploit this, we considered if multiple interactions could be combined to build a series of new POM clusters, while elucidating the mechanism of templated self‐assembly. In addition, an understanding of the structural evolution of the template species may be derived by trapping fragments/intermediates in the larger host structures.

**Scheme 1:**
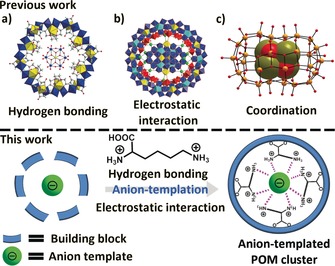
Top: representative interactions such as hydrogen bonding, electrostatic interaction and coordination bonds found between anion templates and POM hosts. Bottom: l‐ornithine as a structure‐directing agent during anion‐templated self‐assembly of Mo Blue clusters, providing both hydrogen bonding (purple dotted line) and electrostatic interactions.

Herein we describe the template‐based self‐assembly of a series of Mo Blue (MB) wheels showing a structural evolution **1**–**4**. These compounds are formed by using l‐ornithine to direct the assembly electrostatically and via hydrogen bonding to stabilize the template⊂host architecture (Scheme 1), giving new mechanistic insights. Compound **1** features a framework of {Mo_124_Ce_4_} with an {Mo_8_} template cluster, while Compound **2** and **3** share the same {Mo_150_Ce_2_} framework, but with different templates: {Mo_17_} for **2** and {PMo_12_} for **3**. Compound **4** comprises of two wheels, {Mo_154_} and {Mo_150_}, with a {Mo_36_} template trapped in the {Mo_150_} framework. Except for {Mo_36_}, the remaining templates are discovered for the first time in MB synthesis and compounds **1**–**4** are totally new clusters in the structural library of MB. The {Mo_17_} unit is isolated for the first time, although its framework has been seen previously in the {Mo_36_} cluster. All compounds were characterized crystallographically and the formulae were determined using an array of techniques (see SI). Compounds **1**–**4** can be formulated as:

$$\left(C{}_{5}H{}_{14}N{}_{2}O{}_{2}\right){}_{2}\left[\left\{\mathrm{Mo}{}_{8}O{}_{26}\right\}{}_{0.5}\subset H{}_{12}\mathrm{Mo}{}_{124}\mathrm{Ce}{}_{4}O{}_{376}\left(H{}_{2}O\right){}_{60}\left(C{}_{5}H{}_{13}N{}_{2}O{}_{2}\right){}_{6}\right]\cdot 160\;H{}_{2}O\;\equiv \;\left(C{}_{5}H{}_{14}N{}_{2}O{}_{2}\right){}_{4}\left[1\;a\right]\cdot 160\;H{}_{2}O\;1$$

$$\mathrm{Na}{}_{3}\left(C{}_{5}H{}_{14}N{}_{2}O{}_{2}\right){}_{2}[\left\{\mathrm{Mo}{}_{17}O{}_{52}\left(H{}_{2}O\right){}_{10}\right\}{}_{0.5}\subset \{H{}_{14}\mathrm{Mo}{}_{150}\mathrm{Ce}{}_{2}O{}_{452}\left(H{}_{2}O\right){}_{76}\left(C{}_{5}H{}_{13}N{}_{2}O{}_{2}\right){}_{6}\}]\cdot 195\;H{}_{2}O\;\equiv \;\mathrm{Na}{}_{3}\left(C{}_{5}H{}_{14}N{}_{2}O{}_{2}\right){}_{2}\left[2\;a\right]\cdot 195\;H{}_{2}O\;2$$

$$\mathrm{Na}{}_{4}H{}_{3}\left(C{}_{5}H{}_{14}N{}_{2}O{}_{2}\right){}_{2}[\left\{\mathrm{PMo}{}_{12}O{}_{40}\right\}\subset \{H{}_{14}\mathrm{Mo}{}_{150}\mathrm{Ce}{}_{2}O{}_{452}\left(H{}_{2}O\right){}_{76}\left(C{}_{5}H{}_{13}N{}_{2}O{}_{2}\right){}_{6}\}]\cdot 195\;H{}_{2}O\;\equiv \;\mathrm{Na}{}_{4}H{}_{3}\left(C{}_{5}H{}_{14}N{}_{2}O{}_{2}\right){}_{2}\left[3\;a\right]\cdot 195\;H{}_{2}O\;3$$

$$\mathrm{Na}{}_{16}\left(C{}_{5}H{}_{14}N{}_{2}O{}_{2}\right){}_{6}[\left\{\mathrm{Mo}{}_{36}O{}_{112}\left(H{}_{2}O\right){}_{16}\right\}\subset \{H{}_{14}\mathrm{Mo}{}_{150}O{}_{452}\left(H{}_{2}O\right){}_{54}\left(C{}_{5}H{}_{13}N{}_{2}O{}_{2}\right){}_{6}\}]\left[H{}_{14}\mathrm{Mo}{}_{154}O{}_{462}\left(H{}_{2}O\right){}_{58}\left(C{}_{5}H{}_{13}N{}_{2}O{}_{2}\right){}_{6}\right]\cdot 410\;H{}_{2}O\equiv \;\mathrm{Na}{}_{16}\left(C{}_{5}H{}_{14}N{}_{2}O{}_{2}\right){}_{6}\left[4\;a{}_{1}\right]\left[4\;a{}_{2}\right]\cdot 410\;H{}_{2}O\;4$$

l‐ornithine was initially introduced into a standard version of the lanthanide‐doped Mo Blue synthesis system (see SI for details),^10^ to establish whether it could assist in trapping an “intrinsic” template that directs the self‐assembly process. Crucially, no preformed anion templates, as established in previous studies, such as the Keggin‐type {M_12_} or Dawson‐type {M_18_} were added, yet crystals of **1** were obtained in two weeks. Single‐crystal X‐ray structure analysis of **1** reveals a host–guest structure **1 a** in which α‐[Mo_8_O_26_]^4−^ ({Mo_8_}) is encapsulated by the {Mo_124_Ce_4_} host (Figure 1).^11^ {Mo_124_Ce_4_} is composed of 12 {Mo_8_} units, 8 {Mo_2_} units, 12 {Mo_1_} units and 4 {Ce(H_2_O)_5_} units functionalized by l‐ornithine ligands on the inner surface. Two Ce^3+^ ions are located on the upper rim and separated by one {Mo_2_} unit while another two are situated on the opposite side and separated by two {Mo_2_} units, giving rise to an elliptical wheel. All the l‐ornithine ligands are attached onto {Mo_2_} units with the side chain buried in the pitch of {Mo_124_Ce_4_}. Due to the limited resolution of the X‐ray data, only three l‐ornithine ligands can be fully located crystallographically. The {Mo_8_} template located at the centre of {Mo_124_Ce_4_} is anchored by a series of N−H⋅⋅⋅O (3.0205(2)–3.7020(2) Å) and C−H⋅⋅⋅O (3.1802(2)‐3.7795(2) Å) hydrogen bonds, which are formed between the terminal O atoms of {Mo_8_} and the methylene and protonated amine groups on the l‐ornithine ligands (Figure S6). In addition, the protonated l‐ornithine also acts as a charge buffer to reduce the repulsive electrostatic force between the anionic {Mo_8_} template and the {Mo_124_Ce_4_} host. The combination of both hydrogen bonding and electrostatic interaction is thus able to stabilize the template⊂host architecture.

**Figure 1:**
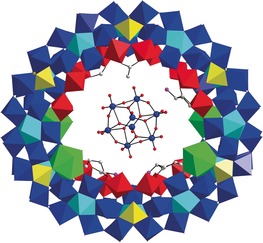
View of the molecular structure of **1 a**. The framework of {Mo_124_Ce_4_} is presented in polyhedron mode and the entrapped {Mo_8_} is shown in ball and stick mode. Color scheme: {Mo_1_}, yellow polyhedron; {Mo_2_}, red polyhedron; {Mo_8_}, blue polyhedron with central pentagonal unit in cyan polyhedron; Ce^3+^, green polyhedron; C, gray ball; N, pink ball.

Under more concentrated reaction conditions, decreasing the ratio of Ce^3+^/MoO_4_^2−^ leads to the discovery of compound **2**. Single crystal X‐ray structure analysis of **2** reveals a host–guest structure **2 a** in which a [Mo_17_O_52_(H_2_O)_10_]^2−^ ({Mo_17_}) is trapped by the {Mo_150_Ce_2_} host (Figure 2 a). The host is isostructural to the {Mo_150_Ce_2_} elliptical ring‐shaped structure that is composed of 14 {Mo_8_} units, 12 {Mo_2_} units, 14 {Mo_1_} units and 2 {Ce(H_2_O)_5_} units, and is functionalized by six protonated l‐ornithine ligands on the inner surface.^12^ The 2 symmetry‐related Ce^3+^ ions are distributed evenly on the two ends of {Mo_150_Ce_2_}, producing an elliptical wheel with outer and inner ring diameter of ≈31 and 12 Å, respectively, at its most elongated points. The six l‐ornithine ligands are grafted onto six {Mo_2_} units via carboxylate groups with the side chain buried in the pitch of {Mo_150_Ce_2_} (Figure 2 a).

**Figure 2:**
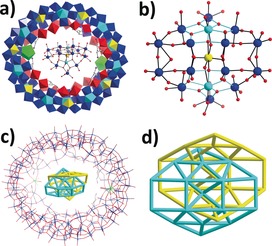
a) View of the molecular structure of **2 a**. {Mo_150_Ce_2_} is shown in polyhedron mode and l‐ornithine is presented in ball and stick mode. Color scheme is the same as Figure 1; b) View of the molecular structure of {Mo_17_}. The pentagon is highlighted using cyan bonds; c) and d) View of the disordered {Mo_17_} in two positions, which are presented in cyan and yellow wire‐frames, respectively.

Similar to **1 a**, the {Mo_17_} template also resides at the centre of {Mo_150_Ce_2_} and is anchored in place by a large number of N−H⋅⋅⋅O (2.9338(1)–3.7231(1) Å) and C−H⋅⋅⋅O (2.9419(1)–3.6863(1) Å) hydrogen bonds formed between the terminal O atoms of {Mo_17_} and the l‐ornithine ligands grafted to the inner surface of the wheel (Figure S7). Moreover, the presence of protonated l‐ornithine ligands minimizes the repulsive electrostatic force between the anionic {Mo_17_} template and {Mo_150_Ce_2_} wheel, thus binding the whole structure together. The {Mo_17_} cluster template is composed of two {Mo_8_} units connected by one {Mo_1_} unit (Figure 2 b). The structural motif of {Mo_17_} could be related to the {Mo_36_} cluster,^13^ which itself can be simplified as two {Mo_17_} joined by two {Mo_1_} units. Previously, the {Mo_36_} cluster has been trapped as a transient template during the self‐assembly of {Mo_154_}9a and, in this regard, {Mo_17_} may therefore be considered as a possible precursor in the formation of the {Mo_36_} template. It should be noted that the {Mo_17_} is disordered equally in two positions within the {Mo_150_Ce_2_} host (Figure 2 c and d).

In our previous work, we have shown that both {PMo_12_} and {P_2_W_18_} can replace the intrinsic {FeMo_6_} template and direct the aggregation of the {Mo_24_Fe_12_} macrocycle.9d Given the formation of an intrinsic or “natural” template in situ for **2**, we considered whether using a preformed template to induce the formation of {Mo_150_Ce_2_} could also be applied here. The addition of preformed {PMo_12_} during the synthesis of **2** resulted in the formation of compound **3**. Consistent with **2**, the single crystal X‐ray structure analysis of **3** reveals a host–guest structure **3 a** where a Keggin‐type anion {PMo_12_} is bound by the host {Mo_150_Ce_2_} (Figure 3 a). The {Mo_150_Ce_2_} host in **3 a** is isostructural to **2 a** but with the {PMo_12_} located in the cavity of {Mo_150_Ce_2_}, in place of {Mo_17_}. As such, similar hydrogen bonding patterns and electrostatic interactions are observed (Figure S8). Due to the relatively smaller size of {PMo_12_} in comparison to the central cavity of {Mo_150_Ce_2_}, the cluster is disordered over two positions, equally, on either side of the framework cavity, maximizing electrostatic interactions and hydrogen bonding (N−H⋅⋅⋅O (2.8850(2)–3.7598(2) Å) and C−H⋅⋅⋅O (3.3208(1)–3.7977(3) Å). Notably, {PMo_12_} adopts the β‐configuration instead of the α‐isomer (Figure 3 b and 3c), due to the β‐form being the preferred species under reduced conditions.^14^

**Figure 3:**
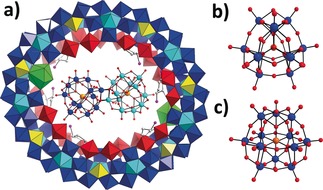
a) View of the molecular structure of **3 a**. The locations of the two disordered {PMo_12_} clusters are presented in blue and cyan, respectively; b) and c) Top view and side view of the entrapped β‐{PMo_12_}. Color scheme: Mo blue, O red, P orange.

The templation effect of encapsulated clusters has been studied in situ by ^31^P NMR spectroscopy. Following the synthetic procedure used to produce compound **3**, after the addition of all starting materials in D_2_O, a ^31^P NMR spectrum was recorded every 10 min for 1 h. When a low concentration of {PMo_12_} (2.5 mg) was used, much less than the stoichiometry required for the formation of {PMo_12_}⊂{Mo_150_Ce_2_}, signals resulting from the reduced {PMo_12_} (−5.69 and −6.25 ppm for β‐{PMo_12_} and α‐{PMo_12_}, respectively) could be detected after 10 min and then completely disappeared after 1 h (Figure S11c,d). Note that the signals of fully oxidized α‐{PMo_12_} are located at −3.35 ppm (Figure S11e). In contrast, the addition of excess {PMo_12_} (8.0 mg) resulted in the presence of peaks of {PMo_12_} throughout the reaction process but the signal became much less intense after 1 h (Figure S11a,b). This indicates that {PMo_12_} is a template during the self‐assembly, which is gradually consumed upon the formation of {PMo_12_}⊂{Mo_150_Ce_2_} (**3 a**). Once {PMo_12_} is included as a template, its ^31^P NMR signal cannot be observed because of the shielding effect of the paramagnetic {Mo_150_Ce_2_}. This is also confirmed by the control experiment where compound **3** showed no ^31^P NMR response in solution (Figure S12). Accordingly, when an inadequate amount of the template was used, the {PMo_12_} is completely encaspulated by the host and hence the {PMo_12_} is NMR silent. When an excess of template is used, this results in the presence of signals even after 1 h due to the remaining presence of free {PMo_12_} (Figure S11a).

The successful encapsulation of {Mo_8_}, {Mo_17_} and {PMo_12_} as templates led us to further explore the potential of using l‐ornithine as a structure‐directing agent to trap different templates, which may in turn give us more insight regarding the formation mechanism of the templated self‐assembly of Mo Blue clusters. In the cases of **1**–**3**, lanthanide‐doped Mo Blue (LMB) {Mo_124_Ce_4_} and {Mo_150_Ce_2_} provide confined environments to enclose templates. In general, LMB‐based wheels exhibit a more curved inner surface and elliptical ring shape, and thus a smaller size compared with the archetypal {Mo_154_} wheel.^15^ With this in mind, we performed the synthesis without adding lanthanides to see how the change of curvature and size of MB will affect the entrapped template. After a systematic optimization of the synthetic conditions we were able to obtain another templated MB cluster **4**, see Figure 4.

**Figure 4:**
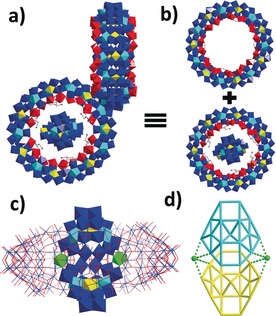
a) and b) View of the molecular structure of **4 a**, consisting of **4 a_1_** and **4 a_2_**; c) Side view of the orientation of {Mo_36_} in {Mo_150_}. {Mo_150_} is presented in wire‐frame mode and the two {Mo_1_} units that connect the two {Mo_17_} units are represented in green polyhedra; d) Simplification of {Mo_36_} to two {Mo_17_} units (cyan and yellow wires) linked by two {Mo_1_} units (green ball).

Single‐crystal X‐ray structure analysis reveals that **4** crystalizes in space group $P\bar{1}$ and consists of two crystallographically independent wheels in the molecular structure, denoted as **4 a_1_** and **4 a_2_**, respectively (Figure 4 a and b). **4 a_1_** features a host–guest architecture with a [Mo_36_O_112_(H_2_O)_16_]^8−^ ({Mo_36_}) trapped by the {Mo_150_} host. The {Mo_150_} adopts the same framework of the archetypal {Mo_154_} that is constructed from 14 sets of {Mo_11_} units, but with two {Mo_2_} defect sites on the rim of the wheel (Figure 4 b).15a The {Mo_36_}, which is situated in the center of the {Mo_150_} cavity, is structurally equivalent to the well‐known and previously described {Mo_36_} cluster,^13^ as observed in the previously reported {Mo_36_}⊂ {Mo_150_}.9a There are also six l‐ornithine ligands attached on six {Mo_2_} units in **4 a_1_**. Among them, two pairs of l‐ornithine are orientated in tail‐to‐tail mode, with the terminal amino groups pointing to each other, while the remaining two are arranged freely.^10^ Similar to **1**–**3**, the positively charged l‐ornithine ligands not only serve as charge buffers to glue the anionic {Mo_36_} template and {Mo_150_} host together, but also form a multiplicity of N−H⋅⋅⋅O (2.7642(1)–3.7894(2) Å) and C−H⋅⋅⋅O (2.7862(1)–3.5290(2) Å) hydrogen bonds with both the terminal and bridging O atoms on {Mo_36_}, to further stabilize the aggregate (Figure S9). In addition, several sodium ions are also found between the {Mo_36_} and {Mo_150_}. Notably, the {Mo_36_} in the previously reported {Mo_36_}⊂{Mo_150_} compound retains a parallel orientation along with {Mo_150_} and is thus completely encased within the cavity of {Mo_150_} (Figure S10).9a However, the {Mo_36_} in **4 a_1_** adopts a vertical orientation that is almost perpendicular to the plane defined by {Mo_150_}, with the two rims stretching out of {Mo_150_} (Figure 4 c). This is caused by the concomitant accommodation of six l‐ornithine in **4 a_1_** and {Mo_36_}, which reduces the available space to fully encompass the {Mo_36_} and thus forces it rotate along its lateral axis, resulting in the {Mo_36_} extending out beyond either side of the {Mo_150_} cavity.

**4 a_2_** adopts the same framework as {Mo_154_} with l‐ornithine functionalizing the inner surface. Due to the limited resolution of crystal data, only the carboxylate group of the l‐ornithine could be identified. Although the central cavity of **4 a_2_** is large enough to accommodate {Mo_36_}, no template is found in the cavity of **4 a_2_**. From the space filling modes of **4 a_1_**, **4 a_2_** and {Mo_36_}⊂{Mo_150_}, it can be seen that the {Mo_150_} host in **4 a_1_** and {Mo_36_}⊂{Mo_150_} exhibits a slightly elliptical ring due to the symmetric defect of two {Mo_2_} units at the two elongated ends, while **4 a_2_** adopts a roughly regular ring as exhibited by the archetypal {Mo_154_} (Figure S10). Taking account of the space occupied by l‐ornithine and the size and shape of {Mo_36_} (1.4 nm×1.6 nm×2.1 nm), this kind of arrangement means the {Mo_150_} can either accommodate {Mo_36_} along its lateral axis (**4 a_1_**) or longitudinal axis ({Mo_36_}⊂{Mo_150_}).In contrast, the cavity of **4 a_2_** is too large in comparison to {Mo_36_} and thus is unable to capture {Mo_36_} efficiently. The encapsulation of {Mo_36_} in **4 a_1_** further confirms that {Mo_36_} is a key template during the self‐assembly of Mo Blue, consistent with our previous study.9a, 9b

The formation of compounds **1**–**4** with l‐ornithine as a structure‐directing agent allows us to propose a potential mechanism of templated self‐assembly underpinning the formation of the compounds, see Scheme 2. The process could be described as follows: firstly, the basic building blocks of {Mo_8_}, {Mo_2_} and {Mo_1_} form in solution. Next, the labile {Mo_8_} units could either transform to the isomeric α‐{Mo_8_} or dimerize with one {Mo_1_} unit to generate the {Mo_17_} cluster in situ, which then behaves as a template to drive the self‐assembly of **1** and **2** under the direction of l‐ornithine, respectively. If the preformed {PMo_12_} is introduced during the synthesis of **2**, then the formation of {Mo_17_} is prevented and **3** will be constructed with {PMo_12_} as the templating species. The dimerization of {Mo_17_} to form {Mo_36_} presents a new template that can direct the formation of **4 a_1_** together with l‐ornithine. The templated self‐assembly revealed here implies that by rationally controlling the sizes, shapes and charges of anionic POM templates with the assistance of structure directors, smaller or larger MB clusters that go beyond the current limitation of sizes and nuclearities can be constructed, leading to the discovery of unprecedented template species and MB framework clusters.

**Scheme 2:**
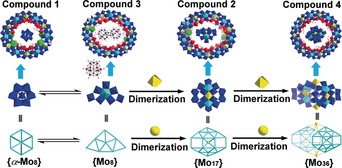
The anion‐templated self‐assembly of macrocycles **1**–**4** and transformation from **2** to **3** by template‐exchange.

In summary, we have described the anion‐templated self‐assembly of MB clusters **1**–**4** featuring template⊂host architectures with l‐ornithine as a structure‐directing agent. Upon protonation, l‐ornithine not only serves as charge buffer to bind the anionic templates and hosts together, but also provides hydrogen bonding sites to strongly interact with templates, which appears to be essential for the formation of **1**–**4**. The successful construction of **1** unveils the “intrinsic template” of {Mo_8_} formed in situ. Under more concentrated conditions, {Mo_17_} is trapped as a template to induce the self‐assembly of **2**, which could be replaced by the preformed {PMo_12_}, leading to the formation of **3**. A ^31^P NMR study during the synthesis of **3** illustrates the self‐assembly is essentially template‐dependent. Moreover, the encapsulation of the {Mo_36_} cluster in **4 a_1_** further confirms that {Mo_36_} is a key template during the self‐assembly of the Mo Blue clusters. Due to the structural relationship between {Mo_8_}, {Mo_17_} and {Mo_36_}, it is reasonable to assume that {Mo_36_} may be constructed via dimerization of {Mo_17_}, which itself can be built from dimerization of {Mo_8_}, showing a potential stepwise pathway for template assembly in solution.

## Conflict of interest

The authors declare no conflict of interest.

## Supporting information

As a service to our authors and readers, this journal provides supporting information supplied by the authors. Such materials are peer reviewed and may be re‐organized for online delivery, but are not copy‐edited or typeset. Technical support issues arising from supporting information (other than missing files) should be addressed to the authors.

Supplementary

## References

[1a] O. Costisor , W. Linert , Metal Mediated Template Synthesis of Ligands, World Scientific Publishing, Singapore, 2004;

[1b] N. V. Gerbeleu , V. B. Arion , J. P. Burgess , Template Synthesis of Macrocyclic Compounds, Wiley-VCH, Weinheim, 1999;

[1c] C. A. Schalley , F. Vögtle , K. H. Dötz , Templates in Chemistry, Springer, Berlin, 2005;

[1d] P. Stang , F. Diederich , Templated Organic Synthesis, Wiley-VCH, Weinheim, 2000;

[1e] Y. Liu , J. Goebl , Y. Yin , Chem. Soc. Rev. 2013, 42, 2610–2653; 23093173 10.1039/c2cs35369e

[1f] C. Liang , Z. Li , S. Dai , Angew. Chem. Int. Ed. 2008, 47, 3696–3717; 10.1002/anie.200702046 18350530

[2a] C. H. Henkels , J. C. Kurz , C. A. Fierke , T. G. Oas , Biochemistry 2001, 40, 2777–2789; 11258888 10.1021/bi002078y

[2b] M. J. Langton , C. J. Serpell , P. D. Beer , Angew. Chem. Int. Ed. 2016, 55, 1974–1987; 10.1002/anie.201506589 PMC4755225 26612067

[2c] H. Barbier-Brygoo , A. D. Angeli , S. Filleur , J.-M. Frachisse , F. Gambale , S. Thomine , S. Wege , Annu. Rev. Plant Biol. 2011, 62, 25–51; 21275645 10.1146/annurev-arplant-042110-103741

[2d] J. L. Sessler , P. A. Gale , W.-S. Cho , Anion Receptor Chemistry, Royal Society of Chemistry, Cambridge, 2006;

[2e] P. Chakrabarti , J. Mol. Biol. 1993, 234, 463–482. 8230226 10.1006/jmbi.1993.1599

[3a] M. S. Vickers , P. D. Beer , Chem. Soc. Rev. 2007, 36, 211–225; 17264924 10.1039/b518077p

[3b] N. Gimeno , R. Vilar , Coord. Chem. Rev. 2006, 250, 3161–3189;

[3c] Supramolecular Chemistry of Anions (Eds.: A. Bianchi, K. Bowman-James, E. Garcia-España), Wiley-VCH, New York, 1997;

[3d] “Themed issue: supramolecular chemistry of anionic species (Eds.: P. A. Gale, T. Gunnlaugson)”: *Chem. Soc. Rev* **2010**, *39*, 3581–4008;

[3e] P. D. Beer , P. A. Gale , Angew. Chem. Int. Ed. 2001, 40, 486–516; 11180358

[4a] “Themed issue: polyoxometalate cluster science (Eds.: L. Cronin, A. Müller)”: *Chem. Soc. Rev* **2012**, *41*, 7325–7648;

[4b] D.-L. Long , E. Burkholder , L. Cronin , Chem. Soc. Rev. 2007, 36, 105–121; 17173149 10.1039/b502666k

[4c] D.-L. Long , R. Tsunashima , L. Cronin , Angew. Chem. Int. Ed. 2010, 49, 1736–1758; 10.1002/anie.200902483 20131346

[4d] D.-Y. Du , L.-K. Yan , Z.-M. Su , S.-L. Li , Y.-Q. Lan , E.-B. Wang , Coord. Chem. Rev. 2013, 257, 702–717;

[4e] M. T. Pope , A. Müller , Polyoxometalate Chemistry: From Topology via Self-Assembly to Applications, Kluwer, Dordrecht, 2001;

[4f] “Special issue on polyoxometalate, (Eds.: C. L. Hill)”: *Chem. Rev* **1998**, *98*, 1–390;

[4g] A. Dolbecq , E. Dumas , C. R. Mayer , P. Mialane , Chem. Rev. 2010, 110, 6009–6048; 20666374 10.1021/cr1000578

[4h] S.-S. Wang , G.-Y. Yang , Chem. Rev. 2015, 115, 4893–4962; 25965251 10.1021/cr500390v

[4i] K. Kamata , K. Yonehara , Y. Sumida , K. Yamaguchi , S. Hikichi , N. Mizuno , Science 2003, 300, 964–966; 12738860 10.1126/science.1083176

[4j] B. Rausch , M. D. Symes , G. Chisholm , L. Cronin , Science 2014, 345, 1326–1330; 25214625 10.1126/science.1257443

[4k] C. Busche , L. Vilà-Nadal , J. Yan , H. N. Miras , D.-L. Long , V. P. Georgiev , A. Asenov , R. H. Pedersen , N. Gadegaard , M. M. Mirza , D. J. Paul , J. M. Poblet , L. Cronin , Nature 2014, 515, 545–549; 25409147 10.1038/nature13951

[4l] M. Shiddiq , D. Komijani , Y. Duan , A. Gaita-Ariño , E. Coronado , S. Hill , Nature 2016, 531, 348–351. 26983539 10.1038/nature16984

[5a] J. F. Keggin , Nature 1933, 131, 908–909;

[5b] B. Dawson , Acta Crystallogr. Sect. B 1953, 6, 113–126;

[5c] R. Strandberg , Acta Chem. Scand. Ser. A 1975, 29, 350–358.

[6a] D.-L. Long , Y. F. Song , E. F. Wilson , P. Kögerler , S. X. Guo , A. M. Bond , J. S. J. Hargreaves , L. Cronin , Angew. Chem. Int. Ed. 2008, 47, 4384–4387; 10.1002/anie.200800041 18442145

[6b] L. Vilà-Nadal , K. Peuntinger , C. Busche , J. Yan , D. Lüders , D.-L. Long , J. M. Poblet , D. M. Guldi , L. Cronin , Angew. Chem. Int. Ed. 2013, 52, 9695–9699; 10.1002/anie.201303126 23873517

[7a] Q. Zheng , L. Vilà-Nadal , C. Busche , J. S. Mathieson , D.-L. Long , L. Cronin , Angew. Chem. Int. Ed. 2015, 54, 7895–7899; 10.1002/anie.201502295 PMC4557056 26013548

[7b] U. Kortz , J. Vaissermann , R. Thouvenot , P. Gouzerh , Inorg. Chem. 2003, 42, 1135–1139. 12588149 10.1021/ic0261427

[8a] M. T. Pope , Heteropoly and Isopoly Oxometalates, Springer, Berlin, 1983;

[8b] J. B. Moffat , Metal-Oxygen Clusters: The Surface and Catalytic Properties of Heteropoly Oxometalates, Springer, New York, 2001;

[8c] X. López , J. J. Carbó , C. Bo , J. M. Poblet , Chem. Soc. Rev. 2012, 41, 7537–7571; 22885565 10.1039/c2cs35168d

[8d] T. Ueda , ChemElectroChem 2018, 5, 823–838;

[8e] Q. Zheng , L. Vilà-Nadal , Z. Lang , J.-J. Chen , D.-L. Long , J. S. Mathieson , J. M. Poblet , L. Cronin , J. Am. Chem. Soc. 2018, 140, 2595–2601; 29359931 10.1021/jacs.7b11982 PMC6075695

[8f] J. Yan , D.-L. Long , E. F. Wilson , L. Cronin , Angew. Chem. Int. Ed. 2009, 48, 4376–4380; 10.1002/anie.200806343 19434635

[8g] U. Kortz , M. G. Savelieff , F. Y. A. Ghali , L. M. Khalil , S. A. Maalouf , D. I. Sinno , Angew. Chem. Int. Ed. 2002, 41, 4070–4073; 10.1002/1521-3773(20021104)41:21<4070::AID-ANIE4070>3.0.CO;2-3 12412084

[9a] H. N. Miras , G. J. T. Cooper , D.-L. Long , H. Bögge , A. Müller , C. Streb , L. Cronin , Science 2010, 327, 72–74; 20044572 10.1126/science.1181735

[9b] H. N. Miras , C. J. Richmond , D.-L. Long , L. Cronin , J. Am. Chem. Soc. 2012, 134, 3816–3824; 22257105 10.1021/ja210206z

[9c] X. Fang , L. Hansen , F. Haso , P. Yin , A. Pandey , L. Engelhardt , I. Slowing , T. Li , T. Liu , M. Luban , D. C. Johnston , Angew. Chem. Int. Ed. 2013, 52, 10500–10504; 10.1002/anie.201304887 23943611

[9d] W. Xuan , R. Pow , D.-L. Long , L. Cronin , Angew. Chem. Int. Ed. 2016, 55, 12703–12707; 10.1002/anie.201603298 PMC5396355 27358195

[9e] A. Müller , S. K. Das , P. Kögerler , H. Bögge , M. Schmidtmann , A. X. Trautwein , V. Schünemann , E. Krickemeyer , W. Preetz , Angew. Chem. Int. Ed. 2000, 39, 3413–3417; 10.1002/1521-3773(20001002)39:19<3413::aid-anie3413>3.0.co;2-q 11091372

[9f] A. Müller , R. Rohlfing , J. Döring , M. Penk , Angew. Chem. Int. Ed. Engl. 1991, 30, 588–590;

[10] W. Xuan , R. Pow , D.-L. Long , L. Cronin , Angew. Chem. Int. Ed. 2017, 56, 9727–9731; 10.1002/anie.201702957 PMC5600119 28508585

[11a] J. Fuchs , H. Hartl , Angew. Chem. Int. Ed. Engl. 1976, 15, 375–376;

[11b] W. G. Klemperer , W. Shum , J. Am. Chem. Soc. 1976, 98, 8291–8293.

[12] T. Yamase , E. Ishikawa , Y. Abe , Y. Yano , J. Alloys Compd. 2006, 408–412, 693–700.

[13a] I. Paulat-Böschen , J. Chem. Soc. Chem. Commun. 1979, 780–782;

[13b] B. Krebs , S. Stiller , K.-H. Tytko , J. Mehmke , Eur. J. Solid State Inorg. Chem. 1991, 28, 883–903.

[14a] X. López , J. M. Maestre , C. Bo , J.-M. Poblet , J. Am. Chem. Soc. 2001, 123, 9571–9576; 11572677 10.1021/ja010768z

[14b] X. López , J.-M. Poblet , Inorg. Chem. 2004, 43, 6863–6865. 15500315 10.1021/ic049119p

[15a] A. Müller , E. Krickemeyer , J. Meyer , H. Bögge , F. Peters , W. Plass , E. Diemann , S. Dillinger , F. Nonnenbruch , M. Randerath , C. Menke , Angew. Chem. Int. Ed. Engl. 1995, 34, 2122–2124;

[15b] L. Cronin , C. Beugholt , E. Krickmeyer , M. Schmidtmann , H. Bögge , P. Kögerler , T. K. K. Luong , A. Müller , Angew. Chem. Int. Ed. 2002, 41, 2805–2808; 10.1002/1521-3773(20020802)41:15<2805::AID-ANIE2805>3.0.CO;2-E 12203492

[15c] A. Müller , C. Beugholt , H. Bögge , M. Schmidtmann , Inorg. Chem. 2000, 39, 3112–3113; 11196845 10.1021/ic000168l

[15d] W. Xuan , A. J. Surman , H. N. Miras , D.-L. Long , L. Cronin , J. Am. Chem. Soc. 2014, 136, 14114–14120; 25188897 10.1021/ja5062483

[15e] A. Müller , P. Gouzerh , Chem. Soc. Rev. 2012, 41, 7431–7463. 22948798 10.1039/c2cs35169b

