# Supplementary material for: Ligand‐Directed Template Assembly for the Construction of Gigantic Molybdenum Blue Wheels
Source: Angew Chem Int Ed Engl. 2019 Jun 28;58(32):10867–72. doi: 10.1002/anie.201901818 (PMC6771582; doi:10.1002/anie.201901818)
Supplement: Supplementary file 1 — Supplementary [file ANIE-58-10867-s001.pdf]

Angewandte Chemie
Eine Zeitschrift der Gesellschaft Deutscher Chemiker

Supporting Information

Ligand-Directed Template Assembly for the Construction of Gigantic Molybdenum Blue Wheels**

*Weimin Xuan, Robert Pow, Qi Zheng, Nancy Watfa, De-Liang Long, and Leroy Cronin**

anie_201901818_sm_miscellaneous_information.pdf

Table of Contents

1. Materials S2

2. Instrumentation S2

3. Synthetic procedure of **1**-**4** S3

4. Structure analysis of **1**-**4** S4

5. Crystallographic data and structure refinement details of **1-4** S12

6. References S21

1. Materials

Reagent-grade chemicals were obtained from Aldrich Chemical Company Ltd. and Alfa Aesar, and used without further purification.

2. Instrumentation

Crystallography: A suitable single crystal was selected and mounted onto a rubber loop using Fomblin oil. X-ray diffraction intensity data was collected using a Bruker Apex II Quasar CCD diffractometer (λ (MoK$_\alpha$) = 0.71073 Å) equipped with a microfocus X-ray source (50 kV, 1.0 mA). Data collection and reduction were performed using the Apex3 software package. Structure solution and refinement were carried out using SHELXS-2016[1] and SHELXL-2016[2] using WinGX suite.[3] Corrections for incident and diffracted beam absorption effects were applied using empirical absorption correction. All the Mo atoms (including those disordered) and most of the O atoms were refined anisotropically. Solvent water molecule sites with partial occupancy were found and included in the structure refinement. Crystallographic formulas typically contain much more water molecules in the crystal lattice than the formulas determined from chemical analyses as a dried sample is used for the latter analyses. It is important to note that with these structures we are moving outside the realm of small molecule crystallography and are dealing with refinements and problems that lie between small molecule and protein crystallography. As a result we cannot expect refinements and statistics to follow the path of crystals with much smaller unit cells. However, the final refinement statistics are relatively good, and in all cases the structural analysis allows us to well determine the structures of the compounds. The X-ray crystallographic data reported in this article have been deposited at the Crystallographic Data Centres. For compounds **1-4**, the data can be obtained free of charge from the Cambridge Crystallographic Data Centre via www.ccdc.cam.ac.uk/data_request/cif under deposition number CCDC-1874914-1874917.

Fourier-transform infrared (FT-IR) spectroscopy: The samples were prepared as a KBr pellet and the FT-IR spectrum was collected in transmission mode in the range of 400-4000 cm^{-1} using a JASCO FT-IR 4100 spectrometer. Wavenumbers are given in cm^{-1}. Intensities are denoted as w = weak, m = medium, s = strong, br = broad, sh = sharp.

Element Analyses: Element analyses for Mo, Ce, K and Na were performed on a Leeman inductivity-coupled plasma (ICP) spectrometer while C, N and H content were determined by the microanalysis

services within the Department of Chemistry, University of Glasgow using an EA 1110 CHNS, CE-440 Elemental Analyzer.

Thermogravimetric Analysis (TGA): Thermogravimetric analysis was performed on a TA Instruments Q 500 Thermogravimetric Analyzer under nitrogen flow at a typical heating rate of 10 °C min^{-1}.

^{31}P Nuclear Magnetic Resonance Spectroscopy: ^{31}P NMR spectroscopy was recorded on a Bruker DPX 500 spectrometer. All samples were prepared by dissolving the clusters in D_2O or performing the reaction directly in D_2O.

3. Synthetic procedure of compounds 1-4

1: $(C_5H_{14}N_2O_2)_2[H_{12}Mo_{128}Ce_4O_{389}(H_2O)_{60}(C_5H_{13}N_2O_2)_6]\cdot 160\ H_2O$, M.W.: 24107.35

L-Ornithine.HCl (6.5 mg, 0.04 mmol), $CeCl_3.7H_2O$ (37.3 mg, 0.1 mmol) and $N_2H_4{\cdot}2HCl$ (4.2 mg, 0.04 mmol) were added to to a solution of $Na_2MoO_4\cdot 2H_2O$ (242 mg, 1 mmol) in water (45 mL). The mixture was then acidified with 1M $HClO_4$ (4.5 mL). After heating with medium stirring in a 100-mL Erlenmeyer flask (widenecked; covered with a watch glass) at 90 °C for 1 h, the resulting clear deep-blue solution was then cooled to room temperature, filtered and kept in an open 100-mL Erlenmeyer flask for 3 weeks. The deep-blue crystals were collected by filtration, washed with ice-cold H_2O, and dried under inert atmosphere over $CaCl_2$, yield: 35 mg (18.6% based on Mo). Elemental analysis, calc. (%): C, 1.99; H, 2.33; N, 0.93; Na, 0; Mo, 50.94; Ce, 2.32; found (%): C, 2.17; H, 1.79; N, 0.91; Na, 0.08; Mo, 50.29; Ce, 2.59. IR (KBr pellet, 4000–400 cm^{-1}): 3386 (s, br), 3180 (s, br), 2922 (w), 2852 (w), 1730 (w), 1608 (s), 1492 (w), 1430 (w), 1350 (w), 967 (m, $\nu_{Mo=O}$), 871 (m), 801 (s), 634 (s), 554 (s).

2: $Na_3(C_5H_{14}N_2O_2)_2[H_{14}Mo_{158.5}Ce_2O_{478}(H_2O)_{81}(C_5H_{13}N_2O_2)_6]\cdot 195H_2O$, M.W.: 29257.07

L-Ornithine.HCl (28.6 mg, 0.17 mmol), $CeCl_3.7H_2O$ (75 mg, 0.2 mmol) and $N_2H_4{\cdot}2HCl$ (15 mg, 0.14 mmol) were added to to a solution of $Na_2MoO_4\cdot 2H_2O$ (1 g, 4.13 mmol) in water (70 mL). The mixture was then acidified with concentrated HCl to pH ~1.0. After heating with medium stirring in a 100-mL Erlenmeyer flask (widenecked; covered with a watch glass) at 90 °C for 1 h, the resulting clear deep-blue solution was then cooled to room temperature, filtered and kept in an open 100-mL Erlenmeyer flask for 1 week. The deep-blue crystals were collected by filtration, washed with ice-cold H_2O, and dried under inert atmosphere over $CaCl_2$, yield: 275 mg (36.4% based on Mo). Elemental analysis, calc. (%): C, 1.64; H, 2.32; N, 0.77; Na, 0.24; Mo, 51.98, Ce, 0.93; found (%): C, 1.66; H, 1.66; N, 0.72; Na, 0.20; Mo, 51.70; Ce, 1.05. IR (KBr

pellet, 4000–400 cm^{-1}): 3396 (s, br), 3184 (s, br), 2921 (w), 2853 (w), 1735 (w), 1611 (s), 1497 (w), 1425 (w), 1341 (w), 968 (m, $\nu_{Mo=O}$), 764 (s), 650 (s), 554 (s).

3: $Na_4H_3(C_5H_{14}N_2O_2)_2[H_{14}Mo_{162}Ce_2PO_{492}(H_2O)_{76}(C_5H_{13}N_2O_2)_6]\cdot195H_2O$, M.W.: 29784.29

L-Ornithine.HCl (28.6 mg, 0.17 mmol), $CeCl_3.7H_2O$ (75 mg, 0.2 mmol), $H_3PMo_{12}O_{40}.nH_2O$ (80 mg, 0.044 mmol) and $N_2H_4\cdot2HCl$ (15 mg, 0.14 mmol) were added to to a solution of $Na_2MoO_4\cdot2H_2O$ (1 g, 4.13 mmol) in water (70 mL). The mixture was then acidified with concentrated HCl to pH ~1.0. After heating with medium stirring in a 100-mL Erlenmeyer flask (widenecked; covered with a watch glass) at 90 °C for 1 h, the resulting clear deep-blue solution was then cooled to room temperature, filtered and kept in an open 100-mL Erlenmeyer flask for 1 week. The deep-blue block-like crystals were collected by filtration, washed with ice-cold H_2O, and dried under inert atmosphere over $CaCl_2$, yield: 256 mg (31.2% based on Mo). Elemental analysis, calc. (%): C, 1.61; H, 2.25; N, 0.75; Na, 0.31; Mo, 52.18; Ce, 0.94; P, 0.10; found (%): C, 1.62; H, 1.59; N, 0.70; Na, 0.30; Mo, 52.40; Ce, 1.08; P, 0.16. IR (KBr pellet, 4000–400 cm^{-1}): 3376 (s, br), 3174 (s, br), 2921 (w), 2852 (w), 1607 (s), 1492 (w), 1432 (w), 1350 (w), 967 (m; $\nu_{Mo=O}$), 867 (m), 766 (s), 634 (s), 554 (s).

4: $Na_{16}(C_5H_{14}N_2O_2)_6[H_{28}Mo_{340}O_{1026}(H_2O)_{128}(C_5H_{13}N_2O_2)_{12}]\cdot410\ H_2O$, M.W.: 61526.30

L-Ornithine.HCl (67.5 mg, 0.40 mmol) and $N_2H_4\cdot2HCl$ (16 mg, 0.15 mmol) were added to to a solution of $Na_2MoO_4\cdot2H_2O$ (3 g, 12.39 mmol) in water (100 mL). The mixture was then acidified with concentrated HCl to pH ~1.2. After heating with medium stirring in a 100-mL Erlenmeyer flask (widenecked; covered with a watch glass) at 90 °C for 1 h, the resulting clear deep-blue solution was then cooled to room temperature, filtered the next day to remove crystalline precipitate and kept in an open 100-mL Erlenmeyer flask for 2 weeks. The deep-blue block-like crystals were collected by filtration, washed with ice-cold H_2O, and dried under inert atmosphere over $CaCl_2$, yield: 260 mg (11.5% based on Mo). Elemental analysis, calc. (%): C, 1.76; H, 2.20; N, 0.82; Na, 0.60; Mo, 53.02; found (%): C, 1.88; H, 1.62; N, 0.82; Na, 0.58; Mo, 51.95. IR (KBr pellet, 4000–400 cm^{-1}): 3409 (s, br), 3223 (s, br), 2923 (w), 2853 (w), 1610 (s), 1493 (w), 1437 (w), 1348 (w), 971 (m; $\nu_{Mo=O}$), 882 (m), 785 (s), 635 (s), 556 (s).

4. Structural analysis of 1-4

Although the wheel-type molybdenum blue architectures are very complex, the general approach to the structural analysis and formula determination is well documented.[4] The structural analysis requires the following lines of evidence / information to allow the assignment of formula and the structural details coupled with single-crystal X-ray diffraction:

(i) Redox titration to help determine the number of reduced Mo^V centres (UV-vis-NIR spectroscopy also can help corroborate this data via the analysis of the extinction coefficient for the LMCT associated with the reduced Mo^V centres. Each centre should contribute ca. 5 - 6 x 10^3 L $mol^{-1}\cdot cm^{-1}$ to ε).
(ii) Bond Valence Sum analysis to confirm the terminal oxo positions, reduced Mo^V centres and the positions of the hydroxide ligands.[5]
(iii) Elemental analysis of sodium, molybdenum, cerium and C, H, N analysis.
(iv) TGA to estimate the number of ligand and solvent water molecules.
Therefore, the analysis below both presents this data and demonstrates how the structural assignment is consistent with this data.

Redox titrations

Because of the rather poor solubility of compounds **1-4**, it is hard to perform redox titration measurmenets to accurately determine the number of reduced metal centers present.

Bond Valence Sum analysis

Table S1. Average bond valence sum values for the Mo centres which span the incomplete $\{Mo_5O_6\}$-type double cubanes and the μ_3-O atoms of the $\{(\mu_3\text{-}O)_2O_2\}$-type compartments in **1**-**4**.

Compounds	BVS (Mo)	BVS (μ_3-O)
**1**	5.71	1.25
**2**	5.59	1.23
**3**	5.58	1.27
**4**	5.56	1.21

Elemental analysis and C, H, N analysis

See Section 3. Synthetic procedure of compounds **1-4**

Uv-vis-NIR spectra and TGA curves

Because of the rather poor solubility of compounds **1-4**, we could not prepare the related solution with accurate concentration. Therefore, the Uv-vis spectra of **1-4** were recorded in saturated aqueous solution and ε was not caculated. All the Uv-vis spectra of **1-4** show the characteristic band of Mo Blue which is centered around 745 nm.

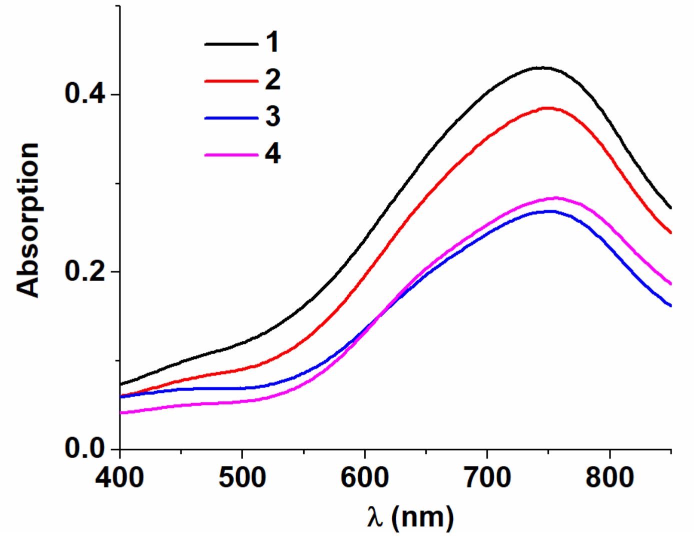

Figure S1 UV-vis-NIR spectra of **1-4** in water.

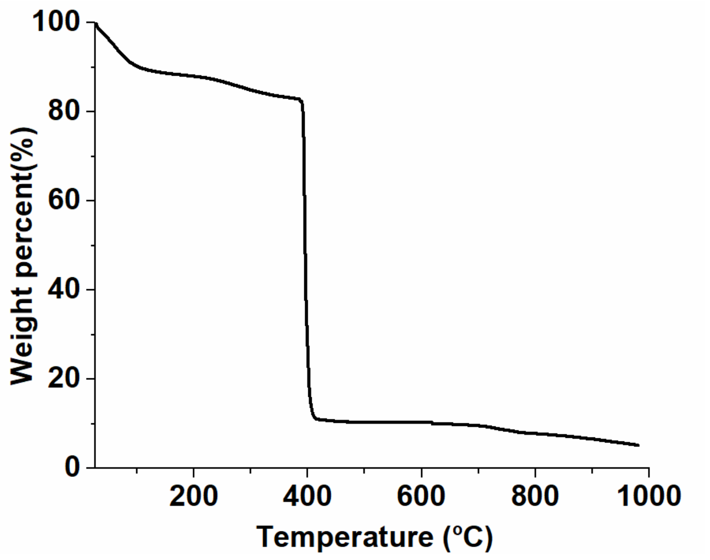

Figure S2 TGA curve for compound **1**. 11.90% weight loss from r.t. to 150 °C corresponds to ~160 H_2O.

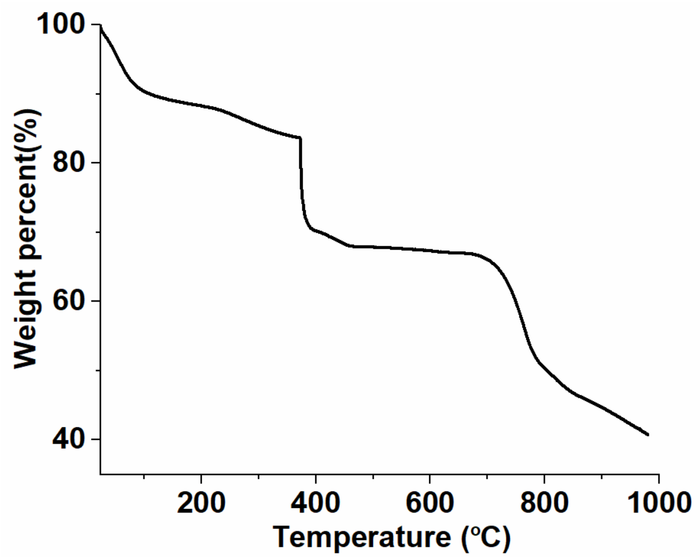

Figure S3 TGA curve for compound **2**. 11.40% weight loss from r.t. to 150 °C corresponds to ~195 H_2O.

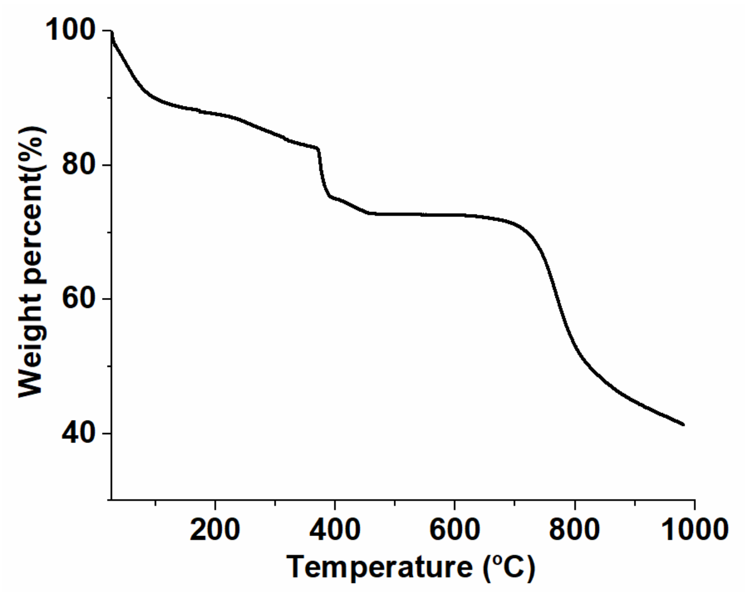

Figure S4 TGA curve for compound **3**. 11.60% weight loss from r.t. to 150 °C corresponds to ~195 H_2O.

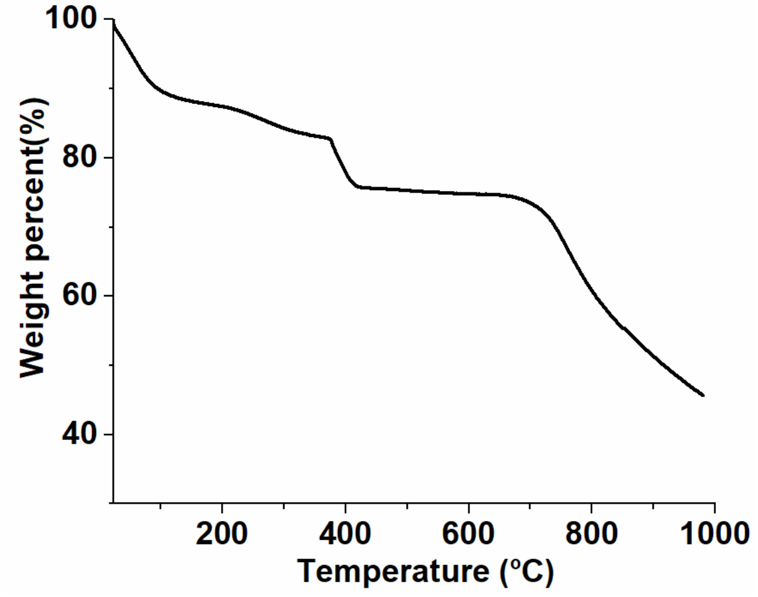

Figure S5 TGA curve for compound **4**. 11.90% weight loss from r.t. to 150 °C corresponds to ~410 H_2O.

Summary of structure analysis

Compound 1 : Firstly, the overall reduction state of **1a** (24 electrons reduced) was confirmed using bond valence sum analysis. The formula of {$Mo_{124}Ce_4$} is calculated based upon the well-established lanthanide-doped Mo Blue. The framework of {$Mo_{124}Ce_4$} could be regarded as 12 sets of {Mo_{11}}, of which four

$\{Mo_2\}$ units are replaced by four $[Ce(H_2O)_5]^{3+}$. The entrapped $\{Mo_8\}$ is highly disordered with an occupancy of 0.5, and the formula is adopted from the classical α-$\{Mo_8\}$ cluster with the formula of $[Mo_8O_{26}]^{4-}$. In the structure solution process, after the $\{Mo_{124}Ce_4\}$ framework was determined, the electron density difference map in cavity of the $\{Mo_{124}Ce_4\}$ ring shows an electron density distribution shape of an α-$\{Mo_8\}$ cluster. Therefore, an ideal α-$\{Mo_8\}$ cluster model in rigid group with fixed occupancy and thermal parameters was introduced to show the cluster position and orientation. Thereafter other partially occupied Mo sites with positions and thermal parameters refined were assigned to define the range of disorders. Occupancy 0.5 of this $\{Mo_8\}$ cluster was estimated based on the occupancy sum of all Mo sites and verified by chemical analysis. There are six doubly protonated L-ornithinate ligands on **1a**. Accordingly, the overall charge of **1a** in **1** is -4. To balance the negative charge of **1a**, two doubly protonated L-ornithine are proposed as counterions for **1a**. Elemental analysis results confirm that the framework of **1a** consists of 128 Mo atoms and four Ce, which is consistent with the single-crystal X-ray diffraction measurement. The amount of L-ornithine is deduced from C, H, N analysis and there are eight L-ornithine in the structure of **1a** in total. Of these, six are located on the framework of **1a** while another two are guest molecules/counterions, as previously stated. The TGA curve of **1** exhibits a total weight loss of 11.4% from r.t. to 150 °C, which corresponds to ~160 guest water molecules. Taking into consideration the information obtained from the above calculations, in addition to single-crystal X-ray diffraction, elemental analyses, bond valence sum analysis and TGA , it is possible to determine the overall building-block scheme and overall formula for **1** as:

$(C_5H_{14}N_2O_2)_4[\{Mo_2\}_8\{Mo_1\}_{12}\{Mo_8\}_{12}(\{Mo_8\}\{Ce\}_4]$ ≡ $(C_5H_{14}N_2O_2)_2[\{Mo^{VI}_2O_5(H_2O)_2\}_8\{Mo^{VI/V}_8O_{26}(\mu_3\text{-}O)_2H(H_2O)_3Mo^{VI/V}\}_{12}\{Mo^{VI}_8O_{26}\}\}\{Ce(H_2O)_5\}_4(C_5H_{13}N_2O_2)_6]ca.160H_2O$

Compounds 2-3 : The formulae of **2** and **3** are determined in a similar manner to **1**. Firstly, the overall reduction state of **2** and **3** (28 and 30 electrons reduced) was confirmed using bond valence sum analysis (BVS) and compared with the archetypal $\{Mo_{154}\}$. Since the encapsulated $\{Mo_{17}\}$ (**2**) and $\{PMo_{12}\}$ (**3**) are partially occupied and disordered, BVS analysis could only be roughly obtained. With aid from chemical and NMR analysis, we concluded that $\{Mo_{17}\}$ (**2**) is not reduced and $\{PMo_{12}\}$ (**3**) is two-electron reduced. In the structure solution process, an ideal model of the $\{Mo_{17}\}$ entity was introduced from the $\{Mo_{17}\}$ moiety contained in a known $\{Mo_{36}\}$ structure. The $\{Mo_{17}\}$ cluster was initially refined as a rigid group with 0.25 occupancy. Then, heavy Mo sites were freed and anisotropically refined but all oxo ligands were still refined as a rigid group. A centro-symmetrically related second part of the $\{Mo_{17}\}$ cluster inside the $\{Mo_{154}\}$ cavity makes up the half occupancy of the $\{Mo_{17}\}$ template in **2**. The β-Keggin $\{PMo_{12}\}$ in **3** was assigned directly and refined with an occupancy of 0.5. A centro-symmetrically related second part of $\{PMo_{12}\}$ cluster inside the $\{Mo_{154}\}$ cavity makes up the full occupancy of the $\{PMo_{12}\}$ template in **2**. There

are six doubly protonated L-ornithinate ligands on **2a** and **3a**. In this way, we could determine the overall charge of **2a** in **2** as -7 and **3a** in **3** as -11. Elemental analysis results confirm that the framework of **2a** consists of 167 Mo atoms and two Ce, and **3a** consists of 162 Mo atoms, two Ce and one P, both of which are consistent with single-crystal X-ray structure determinations. To balance the negative charge of **2a** and **3a**, three sodium ions and two doubly protonated L-ornithine molecules are proposed as counterions for **2a**, and four sodium ions, two doubly protonated L-ornithine and three protons (some attached to the reduced $\{PMo_{12}\}$ cluster) are suggested for **3a,** based on the elemental analysis result of Na. The amount of L-ornithine is deduced from C, H, N analysis and there are eight L-ornithine in the structure of **2a** and **3a** in total. Of these, six are located on the framework of **2a** and **3a** while the remaining two are guest molecules/counterions in solvent areas. Finally, the TGA curves of **2** and **3** exhibit a total weight loss of 11.4% and 11.6% from r.t. to 150 °C, respectively, which corresponds to ~195 guest water molecules.

The archetypal ring $\{Mo_{154}\}$ contains consists of 14 sets of three different building block types: $\{Mo_8\}$, $\{Mo_1\}$ and $\{Mo_2\}$. In a similar way, we can determine the composition of $\{Mo_{150}\}$ in **2a** as $[\{Mo_2\}_{12}\{Mo_1\}_{14}\{Mo_8\}_{14}]^{18-}$ by deleting two $\{Mo_2\}$ units from $\{Mo_{154}\}$. The positions of two further $\{Mo_2\}$ units are replaced by two $[Ce(H_2O)_5]^{3+}$ units. The formula of $\{Mo_{17}\}$ could be evaluated as $[Mo_{17}O_{52}(H_2O)]^{2-}$. Taking into consideration information obtained from the above calculations, in addition to single-crystal X-ray diffraction, elemental analyses, bond valence sum analysis and TGA, it is possible to determine the overall building-block scheme and overall formula for **2** and **3** as:
$Na_3(C_5H_{14}N_2O_2)_2[\{Mo_2\}_{12}\{Mo_1\}_{14}\{Mo_8\}_{14}\{Mo_{17}\}\{Ce\}_2]\equiv Na_3(C_5H_{14}N_2O_2)_2[\{Mo^{VI}{}_2O_5(H_2O)_2\}_{12}$ $\{Mo^{VI/V}{}_8O_{26}(\mu_3O)_2H(H_2O)_3Mo^{VI/V}\}_{14}(C_5H_{13}N_2O_2)_6\{Mo_{17}O_{52}(H_2O)_{10}\}\{Ce(H_2O)_5\}_2]\cdot ca.195H_2O$ (**2**)

$Na_4H_3(C_5H_{14}N_2O_2)_2[\{Mo_2\}_{12}\{Mo_1\}_{14}\{Mo_8\}_{14}\{PMo_{12}\}\{Ce\}_2]\equiv$ $Na_4H_3(C_5H_{14}N_2O_2)_2[\{Mo^{VI}{}_2O_5(H_2O)_2\}_{12}$ $\{Mo^{VI/V}{}_8O_{26}(\mu_3O)_2H(H_2O)_3Mo^{VI/V}\}_{14}(C_5H_{13}N_2O_2)_6\{PMo_{12}O_{40}\}\{Ce(H_2O)_5\}_2]\cdot ca.195H_2O$ (**3**)

Compound 4 : The formula of **4** is determined in a similar manner to **1**. Firstly, the overall reduction state of **4a** (28 electrons reduced) was confirmed using bond valence sum analysis. The formula of the $\{Mo_{154}\}$ wheel (**4a₂**) is adopted directly from archetypal ring $\{Mo_{154}\}$, while $\{Mo_{150}\}$ (**4a₁**) is derived by removing two $\{Mo_2\}$ units from the same archetypal $\{Mo_{154}\}$ structure. A trapped $\{Mo_{36}\}$ cluster, similar to the previously reported $\{Mo_{36}\}$, was found inside the $\{Mo_{150}\}$ cavity. There are six doubly protonated L-ornithinate ligands on **4a₁** and **4a₂**. Accordingly, the overall charge of **4a₁** and **4a₂** in **4** is -28. Elemental analysis indicated the presence of 340 Mo on the frameworks of **4a₁** and **4a₂**. To balance the negative charge of **4a₁** and **4a₂**, 16 sodium ions and 6 doubly protonated L-ornithine are proposed as counterions. The

number of L-ornithine present is deduced from C, H, N analysis, which indicates that there are in total 18 L-ornithine in the structure of **4**. Among them, six are located on the framework of **4a$_1$** and **4a$_2$**, respectively, while another six are guest molecules/counterions. TGA curve of **4** exhibits a total weight loss of 11.9% from r.t. to 150 °C, which corresponds to ~410 guest water molecules.

Taking into consideration the information obtained from the above calculations, in addition to single-crystal X-ray diffraction, elemental analyses, bond valence sum analysis and TGA, it is possible to determine the overall building-block scheme and overall formula for **4** as:

$Na_{16}(C_5H_{14}N_2O_2)_6[\{Mo_2\}_{12}\{Mo_1\}_{14}\{Mo_8\}_{14}\{Mo_{36}\}][\{Mo_2\}_{14}\{Mo_1\}_{14}\{Mo_8\}_{14}]$ ≡ $Na_{16}(C_5H_{14}N_2O_2)_6$ $[\{(Mo^{VI}{}_2O_5)_{12}(H_2O)_{12}(C_5H_{13}N_2O_2)_6\}\{Mo^{VI/V}{}_8O_{26}(\mu_3\text{-}O)_2H(H_2O)_3Mo^{VI/V}\}_{14}\{Mo^{VI}{}_{36}O_{112}(H_2O)_{16}\}]$ $[\{(Mo^{VI}{}_2O_5)_{14}(H_2O)_{16}(C_5H_{13}N_2O_2)_6\}\{Mo^{VI/V}{}_8O_{26}(\mu_3\text{-}O)_2H(H_2O)_3Mo^{VI/V}\}_{14}]\cdot ca.410H_2O$

5. Crystallographic data and structure refinement details of 1-4

Table S2. Crystal data and structure refinement for **1**

Identification code	**1**
Empirical formula	$C_{40}H_{558}Ce_4Mo_{128}N_{16}O_{625}$
Formula weight	24107.79
Temperature (K)	150(2)
Wavelength (Å)	0.71073
Crystal system	Orthorhombic
Space group	$P2_12_12_1$
Unit cell dimensions	a = 38.114(3), α = 90º
	b = 39.898(3), β = 90º
	c = 47.356(3), γ = 90º
Volume (Å^3), Z	72014(8), 4
Density (calculated) (mg/m^3)	2.224
Absorption coefficient (mm^{-1})	2.499
F(000)	46072
Crystal size (mm^3)	0.100 x 0.050 x 0.050
θ range for data collection (º)	2.137 to 26.000
Limiting indices	-47<=h<=47, -49<=k<=49, -58<=l<=58
Reflections collected	918265
Independent reflections	141437 [R(int) = 0.0511]
Completeness to theta	25.242/ 99.9%
Absorption correction	Empirical
Max. and min. transmission	0.745 and 0.675
Refinement method	Full-matrix least-squares on F^2
Data / restraints / parameters	192434 / 46 / 8866
Goodness-of-fit on F^2	1.172
Final R indices [I>2sigma(I)]	R1 = 0.0693, wR2 = 0.1754
R indices (all data)	R1 = 0.0937, wR2 = 0.2150
Absolute structure parameter	0.005(3)
Largest diff. peak and hole (e.Å^{-3})	2.36 and -1.84

Table S3. Crystal data and structure refinement for **2**

Identification code	**2**
Empirical formula	$C_{40}H_{672}Ce_2Mo_{158.50}N_{16}Na_3O_{770}$
Formula weight	29257.60
Temperature (K)	150(2)
Wavelength (Å)	0.71073
Crystal system	Orthorhombic
Space group	*P*bca
Unit cell dimensions	a = 36.1134(15), α = 90º
	b = 47.727(2), β = 90º
	c = 47.799(2), γ = 90º
Volume ($Å^3$), Z	82387(6), 4
Density (calculated) (mg/m^3)	2.359
Absorption coefficient (mm^{-1})	2.545
F(000)	55960
Crystal size (mm^3)	0.100 x 0.050 x 0.050
θ range for data collection (º)	1.022 to 25.382
Limiting indices	-43<=h<=42, -56<=k<=57, -57<=l<=49
Reflections collected	557824
Independent reflections	75410 [R(int) = 0.0891]
Completeness to theta	25.242/ 99.9%
Absorption correction	Empirical
Max. and min. transmission	0.7452 and 0.6056
Refinement method	Full-matrix least-squares on F^2
Data / restraints / parameters	75410 / 30 / 3853
Goodness-of-fit on F^2	1.245
Final R indices [I>2sigma(I)]	R1 = 0.0920, wR2 = 0.2063
R indices (all data)	R1 = 0.1473, wR2 = 0.2683
Largest diff. peak and hole (e.$Å^{-3}$)	2.13 and -1.47

Table S4. Crystal data and structure refinement for **3**

Identification code	**3**
Empirical formula	$C_{40}H_{665}Ce_{2}Mo_{162}N_{16}Na_{4}O_{779}P$
Formula weight	29784.29
Temperature (K)	150(2)
Wavelength (Å)	0.71073
Crystal system	Orthorhombic
Space group	*P*bca
Unit cell dimensions	a = 47.732(4), α = 90º
	b = 36.019(3), β = 90º
	c = 47.894(4), γ = 90º
Volume ($Å^3$), Z	82343(12), 4
Density (calculated) (mg/m^3)	2.403
Absorption coefficient (mm^{-1})	2.602
F(000)	56912
Crystal size (mm^3)	0.100 x 0.050 x 0.050
θ range for data collection (º)	1.107 to 25.204
Limiting indices	57<=h<=57, -43<=k<=40, -44<=l<=57
Reflections collected	565504
Independent reflections	73917 [R(int) = 0.0900]
Completeness to theta	25.204 / 99.6%
Absorption correction	Empirical
Max. and min. transmission	0.745 and 0.597
Refinement method	Full-matrix least-squares on F^2
Data / restraints / parameters	73917 / 24 / 4076
Goodness-of-fit on F^2	1.255
Final R indices [I>2sigma(I)]	R1 = 0.0850, wR2 = 0.1821
R indices (all data)	R1 = 0.1217, wR2 = 0.2115
Largest diff. peak and hole ($e.Å^{-3}$)	2.35 and -1.86

Table S5. Crystal data and structure refinement for **4**

Identification code	**4**
Empirical formula	$C_{90}H_{1344}Mo_{340}N_{36}Na_{16}O_{1600}$
Formula weight	61527.37
Temperature (K)	150(2)
Wavelength (Å)	0.71073
Crystal system	Triclinic
Space group	*P*-1
Unit cell dimensions	a = 27.367(4) , α = 98.600(7)°
	b = 27.663(4) , β = 98.834(7)°
	c = 63.835(8) , γ = 94.197(7)°
Volume ($Å^3$), Z	46987(11), 1
Density (calculated) (mg/m^3)	2.174
Absorption coefficient (mm^{-1})	2.291
F(000)	29392
Crystal size (mm^3)	0.100 x 0.050 x 0.050
θ range for data collection (°)	0.766 to 24.831
Limiting indices	-32<=h<=31, -32<=k<=32, -74<=l<=68
Reflections collected	493374
Independent reflections	156210 [R(int) = 0.1206]
Completeness to theta	24.831/ 96.2%
Absorption correction	Empirical
Max. and min. transmission	0.745 and 0.488
Refinement method	Full-matrix least-squares on F^2
Data / restraints / parameters	156210 / 190 / 7678
Goodness-of-fit on F^2	1.079
Final R indices [I>2sigma(I)]	R1 = 0.1150, wR2 = 0.2793
R indices (all data)	R1 = 0.1921, wR2 = 0.3416
Largest diff. peak and hole (e.$Å^{-3}$)	2.05 and -2.02

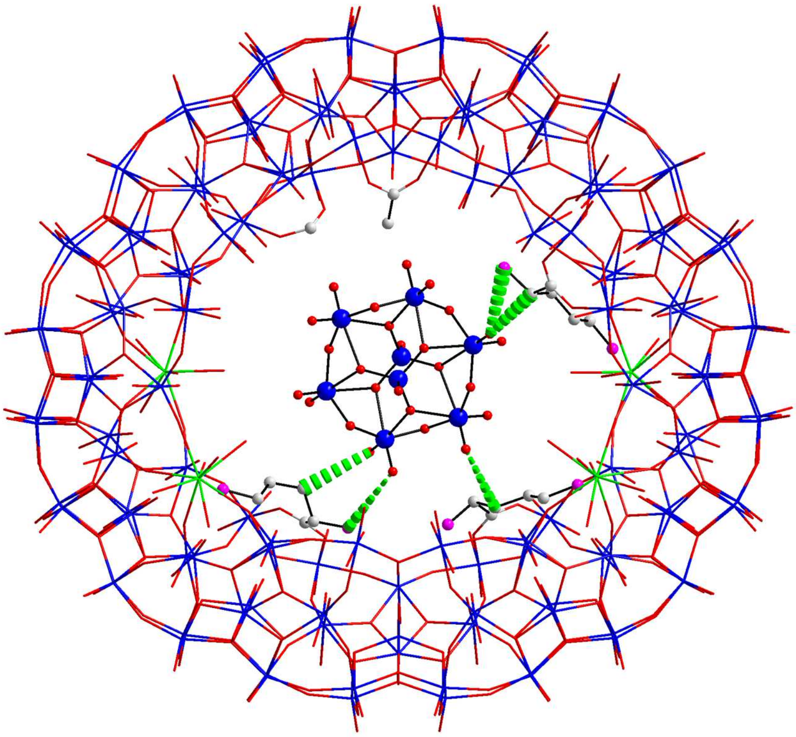

Figure S6. Hydrogen bonds formed between {Mo_8} and L-ornithine in **1a**. Mo, blue; Ce, green; O, red; C, gray; N, pink.

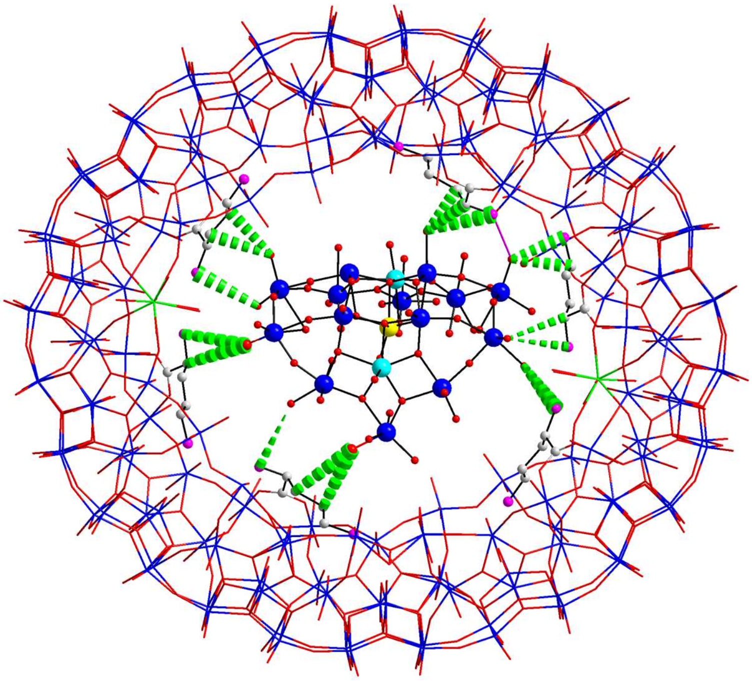

Figure S7. Hydrogen bonds formed between {Mo_{17}} and L-ornithine in **2a**. Mo, blue; Ce, green; O, red; C, gray; N, pink.

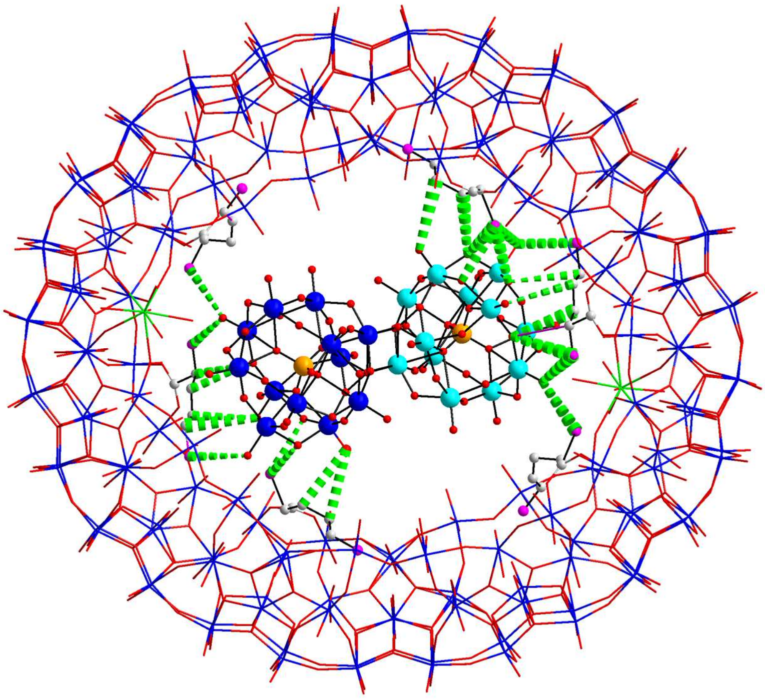

Figure S8. Hydrogen bonds formed between {PMo_{12}} and L-ornithine in **3a**. Mo, blue; Ce, green; O, red; C, gray; N, pink.

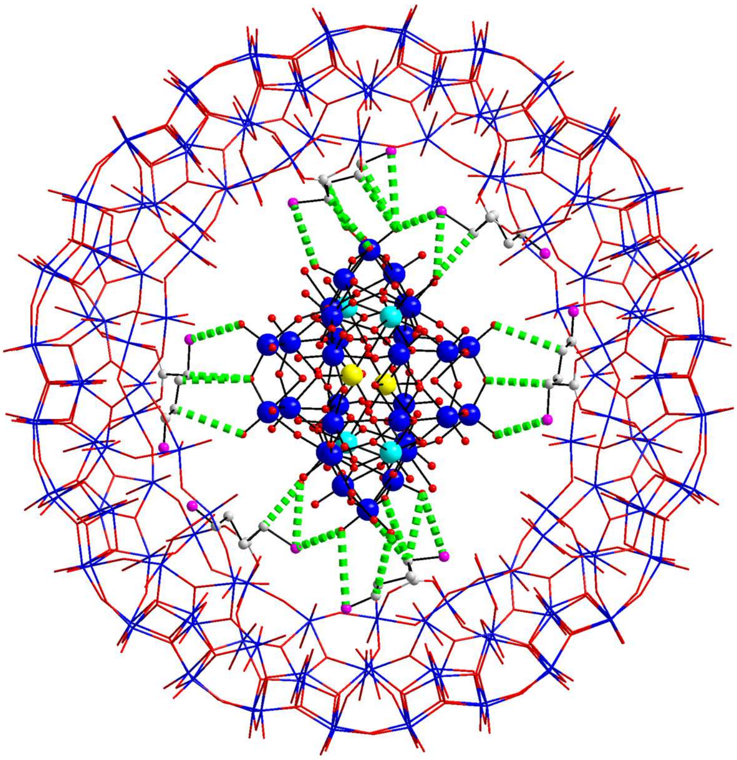

Figure S9. Hydrogen bonds formed between {Mo_{36}} and L-ornithine in **4a₁**. Mo, blue; Ce, green; O, red; C, gray; N, pink.

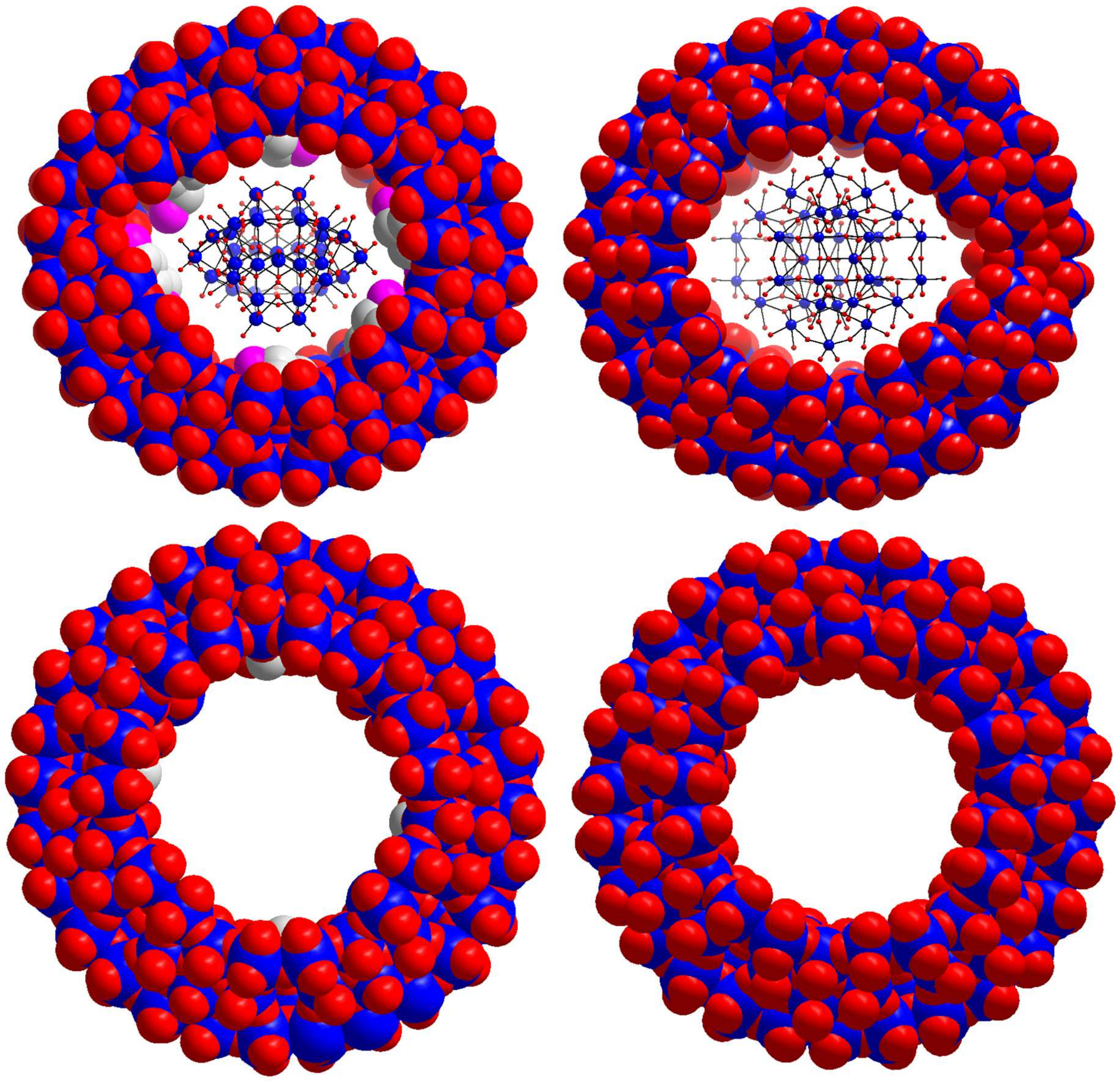

Figure S10. Space filling mode of $\mathbf{4a_1}$ (top left), $\{Mo_{36}\}@\{Mo_{150}\}$(top right),[6] $\mathbf{4a_2}$ (bottom left) and $\{Mo_{154}\}$ (bottom right). The $\{Mo_{36}\}$in $\mathbf{4a_1}$ and $\{Mo_{36}\}@\{Mo_{150}\}$[6] is presented in ball and stick mode. Mo, blue; Ce, green; O, red; C, gray; N, pink.

5. Time resolved ^{31}P NMR study of the templated assembly of 3

The time-resolved ^{31}P NMR study is performed during the scaledown synthesis of **3**:

L-Ornithine.HCl (2.87 mg, 0.017 mmol), $CeCl_3.7H_2O$ (7.5 mg, 0.02 mmol), $H_3PMo_{12}O_{40}.nH_2O$ (8 mg, 0.0044 mmol) and $N_2H_4{\cdot}2HCl$ (1.5 mg, 0.014 mmol) were added to to a solution of $Na_2MoO_4{\cdot}2H_2O$ (0.1 g, 0.413 mmol) in mixture of water (5 mL) and D_2O (1 mL). The mixture was then acidified with 2M HCl to pH ~1.0 and transferred to NMR tube equipped with an inserted tube that contains phenyl phosphonic acid (2 mg) in D_2O as external reference. The NMR tube was then heated at 70 °C for 1 h, and the ^{31}P NMR spectrum was recorded every 10 min (Figure S10a and S10b). **Note:** the chemical shift of phenyl phosphonic acid is at 16.325 ppm.

To make a comparison, 2.5 mg $H_3PMo_{12}O_{40}.nH_2O$ was used for ^{31}P NMR study in the same way (Figure S10c and S10d). Since the theoretical amount of $H_3PMo_{12}O_{40}.nH_2O$ that is required to template the self-assembly of all the $Na_2MoO_4{\cdot}2H_2O$ (0.1 g) into $\{Mo_{150}Ce_2\}$ (0.0027 mmol) is around 5 mg. In principle, 2.5 mg $H_3PMo_{12}O_{40}.nH_2O$ will be completely consumed as template while excess $H_3PMo_{12}O_{40}.nH_2O$ will remain in solution when 8 mg is used. Therefore, the ^{31}P NMR signal corresponding to $H_3PMo_{12}O_{40}.nH_2O$ should be still observed after the reaction when using 8 mg $H_3PMo_{12}O_{40}.nH_2O$ whereas no signal should be seen in the case of 2.5 mg $H_3PMo_{12}O_{40}.nH_2O$ (Figure S11a-d).

The ^{31}P NMR spectrum of $H_3PMo_{12}O_{40}.nH_2O$ $\{PMo_{12}\}$ (Figure S11e) was recorded by dissolving 4 mg $H_3PMo_{12}O_{40}.nH_2O$ in 0.5 mL D_2O with pH adjusted to 1.0 by 2M HCl. In the same way, phenyl phosphonic acid (2 mg) was used as external reference.

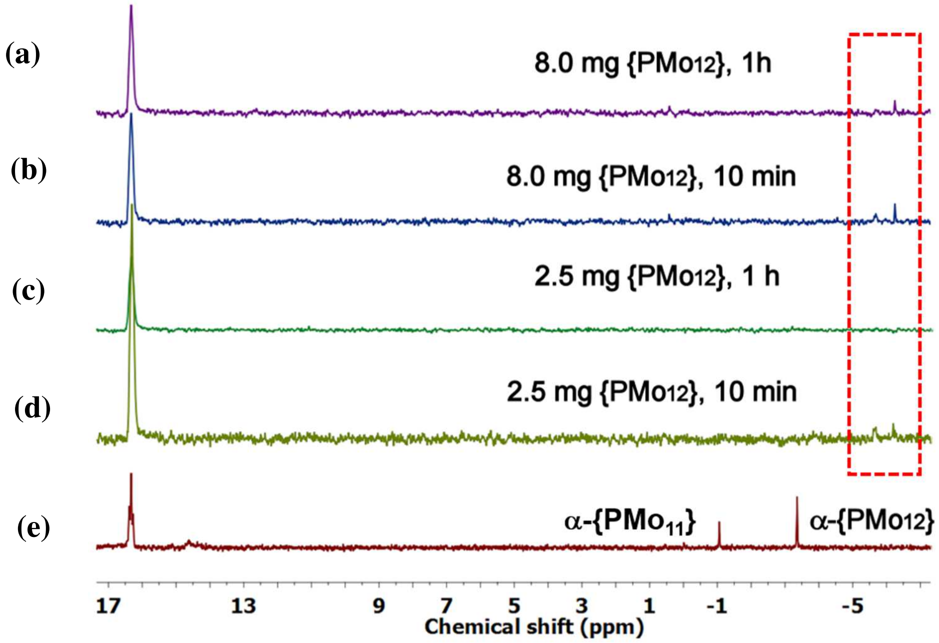

Figure S11. ^{31}P NMR study of the templated assembly of **3.** From top to bottom, (a) 8.0 mg $\{PMo_{12}\}$ reacting for 1 h; (b) 8.0 mg $\{PMo_{12}\}$ reacting for 10 min; (c) 2.5 mg $\{PMo_{12}\}$ reacting for 1 h; (d) 2.5 mg $\{PMo_{12}\}$ reacting for 10 min; (e) 4 mg $\{PMo_{12}\}$ only. The signal at 16.32 ppm corresponds to the external reference, phenyl phosphonic acid. The signals corresponding to α-$\{PMo_{12}\}$ and α-$\{PMo_{12}\}$ are highlighted in red dotted rectangle.

The NMR study of compound **3** was also performed as a control experiment. The solution of **3** was prepared as follows: **3** (40 mg, 0.0027 mmol) was added to to a mixture of water (2.5 mL) and D_2O (0.5 mL). The mixture was then acidified with 2M HCl to pH ~1.0 and sonicated for 0.5 h to aid dissolution. The resulting blue solution was filtered to remove any solid and transferred to an NMR tube equipped with an axial reference tube that contained phenyl phosphonic acid (4 mg) in D_2O as external reference. The NMR spectrum of **3** shows the peak of phenyl phosphonic acid only and no signal can be detected for $H_3PMo_{12}O_{40}$ as this cluster is entraped by the $\{Mo_{150}Ce_2\}$ host in **3**.

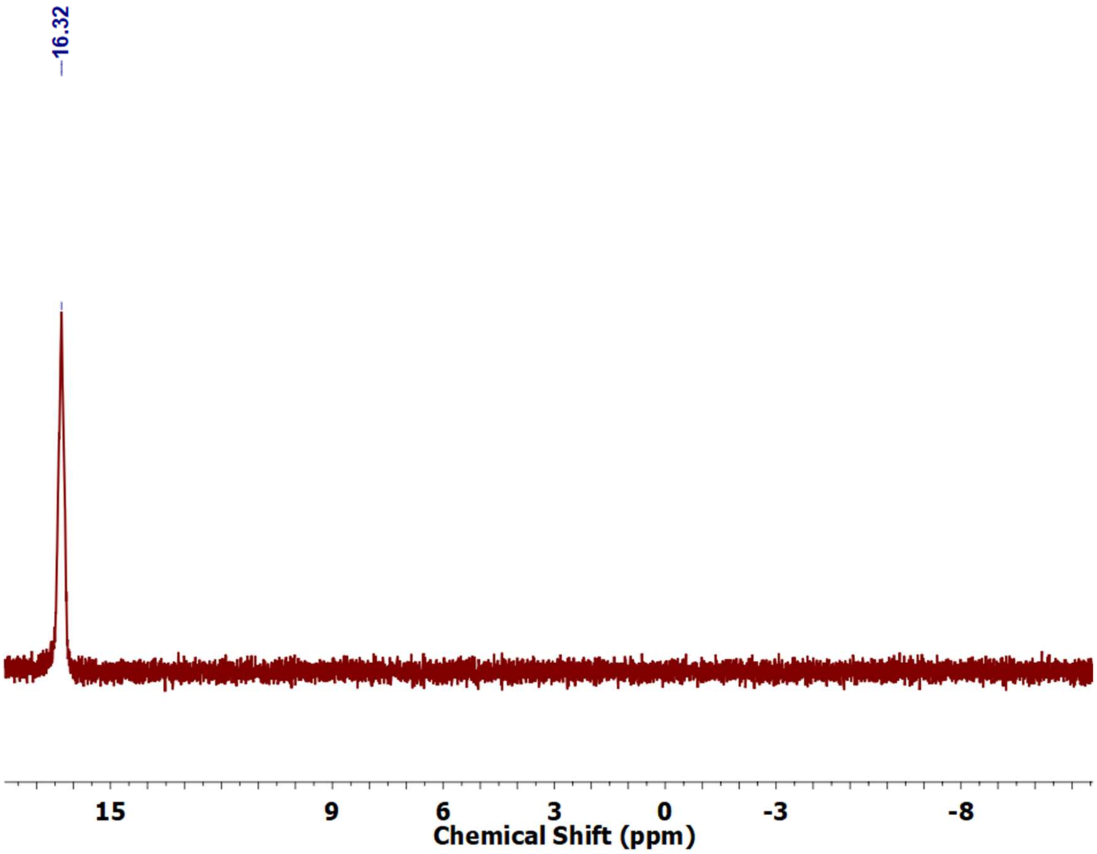

Figure S12. Controled ^{31}P NMR spectrum of **3** in a mixed D_2O and H_2O solvent system**.** The conditions and parameter settings are identical to the spectra collected for the samples in Figure S11. A single signal is observed at 16.32 ppm which corresponds to the external reference only, phenyl phosphonic acid.

6. Reference

1. G. Sheldrick, *ActaCrystallographica Section A*, **1990**, *46*, 467-473.
2. G. Sheldrick, *ActaCrystallographica Section A*, **2008**, *64*, 112-122.
3. L. Farrugia, *J. Appl. Crystallogr.*, **1999**, *32*, 837-838.
4. a) A. Müller, E. Krickemeyer, H. Bögge, M. Schmidtmann, C. Beugholt, S. K. Das, F. Peters, *Chem. Eur. J.* **1999***,* 5, 1496-1502; b) A. Müller, C. Serain, *Acc. Chem. Res.* **2000,** *33*, 2-10.
5. I. D. Brown, In Structure and Bonding in Crystals, Vol. II (Ed.: M.O'Keefe, A. Navrotdsky, Academic Press, New York, 1981), p. 1.
6. H. N. Miras, G. J. T. Cooper, D.-L. Long, H. Bögge, A. Müller, C. Streb, L. Cronin, Science 2010, 327, 72–74.
